# VISTA is associated with immune infiltration and predicts favorable prognosis in TNBC

**DOI:** 10.3389/fonc.2022.961374

**Published:** 2022-09-08

**Authors:** Mi Zhang, Juan Zhang, Na Liu, Biyuan Wang, Yan Zhou, Jin Yang

**Affiliations:** Department of Medical Oncology, The First Affiliated Hospital of Xi’an Jiaotong University, Xi’an, China

**Keywords:** VISTA, triple-negative breast cancer (TNBC), prognosis, immune infiltration, M1 macrophages

## Abstract

**Background:**

V-domain Ig-containing suppressor of T cell activation (VISTA), a critical immune checkpoint protein, can regulate the immune system. Nevertheless, little information is available on the expression level of VISTA and its clinical significance as well. The immunological and prognostic role of VISTA in triple-negative breast cancer (TNBC) still remains unclear.

**Methods:**

The clinical significance and expression of VISTA in TNBC were examined using RNA sequencing and clinical data. Cancer single-cell state atlas (CancerSEA), gene set enrichment analyses (GSEA), single sample GSEA, ESTIMATE algorithm, immunohistochemistry (IHC) were utilized to assess the functions of VISTA.

**Results:**

VISTA was down-regulated and closely associated with good prognosis in TNBC. The expression of VISTA was higher in Immunity-H group and immunomodulatory (IM) subtype. The level of VISTA expression in TNBC gradually increased with the degree of stromal tumor infiltrating lymphocytes (sTILs) infiltration. In addition, the high expression of VISTA was strongly linked to higher proportion of CD8 (+) T cell and M1 macrophages.

**Conclusion:**

VISTA was remarkably correlated with a favorable prognosis and high immune infiltration in patients with TNBC.

## Introduction

Breast cancer (BC) is the most common cancer in women worldwide (1). Triple-negative breast cancer (TNBC) is histologically defined by the absence of the expression of estrogen receptor, progesterone receptor, and epidermal growth factor receptor-2 (HER-2). TNBC is a highly aggressive subtype of BC with a high incidence of local recurrence and metastasis. It accounts for approximately 15% of all types of BC (2). Therefore, in the last decades, endocrine therapy and anti-HER-2 therapy were not recommended, chemotherapy was the only available systemic treatment option for TNBC patients.

In recent years, immunotherapies based on immune checkpoint-blocking antibodies have achieved some success. TNBC is the most immunogenic subtype of BC. The introduction of immunotherapy into the treatment of TNBC has produced survival benefits. Programmed cell death ligand 1 (PD-L1) blocking antibody (atezolizumab) has been approved by European Medicines Agency and Food and Drug Administration for PD-L1 positive metastatic TNBC (3), because the first-line treatment with atezolizumab plus nab-paclitaxel can improve survival. Nevertheless, the response rate to immune checkpoint inhibitors (ICIs) is low. In the United States, less than 13% of patients with cancer benefit from the immunotherapy (4). In metastatic TNBC, the objective response rates (ORR) of ICIs, as a monotherapy, do not exceed 24% (5). Additionally, some cancer patients with initial response to ICIs would develop acquired resistance later (6). Despite receiving continued therapy, about one-quarter to one-third of metastatic melanoma patients who have objective response to ICIs therapies eventually relapsed (7). Based on above reasons, the sustained benefits of immunotherapy have been limited to a small number of patients. Hence, there is an urgent need to explore new targets.

As a type I immunoglobulin membrane protein, V-domain Ig-containing suppressor of T cell activation (VISTA) exerts immunosuppressive activities on T cells and plays a crucial role in the regulation of antitumor immunity (8–11). Relevant studies have shown that VISTA inhibits T cell activation through T cell-extrinsic and intrinsic mechanisms. On the one hand, VISTA expressed on antigen presenting cells (APCs) may act as a ligand that binds to inhibitory receptors. On the other hand, VISTA expressed on T cells may act as a receptor that transmits inhibitory signals (12). Although both VISTA and programmed cell death receptor 1 (PD-1) are immune checkpoint proteins that inhibit T cell activation, VISTA and PD-L1/PD-1 pathways independently control tumor-specific T-cell responses (13). Blocking VISTA and PD-L1 in murine tumor models can achieve synergistic therapeutic efficacy and enhance antitumor responses. Besides, the combined inhibition of cytotoxic T-lymphocyte-associated protein 4 (CTLA-4) and VISTA is more efficacious, and could increase CD8/Treg and Tcon/Treg ratios in the tumor microenvironment (TME) (14). In conclusion, more and more evidence indicated that VISTA is a new emerging target in cancer immunotherapy.

Many studies have proved that VISTA is a potential prognostic factor, while their conclusions are controversial. Villarroel et al. revealed that the high level of VISTA measured in the tumor area was significantly associated with better 5-year overall survival (OS) in non-small-cell lung cancer (NSCLC) (15). Coincidentally, Loeser et al. found that VISTA positive tumors showed a major survival advantage in early-stage esophageal adenocarcinoma (pT1/2) compared with VISTA negative tumors (16). In contrast, Kuklinski et al. suggested that high VISTA expression was remarkably correlated with worse disease-specific survival (DSS) in primary cutaneous melanoma (17). To date, there are few studies on VISTA in TNBC. The clinical significance of VISTA, and its potential role and mechanism in tumor immune infiltration in patients with TNBC still remain elusive.

In this study, multiple databases were used to examine the expression, clinical correlates, and prognostic significance of VISTA in TNBC patients. Moreover, gene set enrichment analyses (GSEA), single sample GSEA, ESTIMATE algorithm, immunohistochemistry (IHC) and multiplex immunofluorescence (mIF) were utilized to explore the potential biological functions of VISTA. Our study aimed to shed light on the role of VISTA in prognosis and tumor immune infiltration of TNBC.

## Materials and methods

### Data acquisition

TNBC and para-carcinoma tissue datasets were acquired from The Cancer Genome Atlas (TCGA) (https://portal.gdc.cancer.gov/). A total of 11 TNBC samples from TCGA were included in our study. RNA sequencing and clinical data were obtained from 359 TNBC patients collected at the Fudan University Shanghai Cancer Center (FUSCC) (FUSCC cohort) (18). The clinical data included age, gender, histology, tumor T stage, N stage and stromal tumor infiltrating lymphocytes (sTILs). The RNA sequencing data of 131 TNBC patients from GSE83937 (https://www.ncbi.nlm.nih.gov/geo/) were adopted to verify the main findings from the FUSCC cohort.

### Kaplan-Meier plotter

The Kaplan-Meier plotter database (http://kmplot.com/analysis) was used to evaluate the prognostic value of VISTA expression in TNBC.

### Breast cancer integrative platform

The BCIP (http://www.omicsnet.org/bcancer/) is an integrative platform. The Molecular Taxonomy of Breast Cancer International Consortium (METABRIC) database acquired from the BCIP was used to analyze the relationship between VISTA expression and OS in TNBC.

### GSEA

In order to investigate biological function and potential signaling pathway of VISTA expression level in tumor tissues, GSEA was carried out using TPM of RNA sequencing data from the FUSCC cohort.

### CancerSEA

CancerSEA (http://biocc.hrbmu.edu.cn/CancerSEA/home.jsp) involves 14 functional states of 41900 cancer single cells from 25 tumor types (19). In this work, it was used to evaluate the functional state of VISTA in BC.

### CIBERSORT

CIBERSORT (20) is an algorithm to determine the possible proportion of immune cells in a sample based on gene expression profile.

### Landscape of immune infiltration status in TNBC

The single sample GSEA was performed to quantify the relative abundance of each cell infiltration in the TNBC tumor immune microenvironment (TIME). The enrichment scores of all samples in two cohorts (FUSCC and Gene Expression Omnibus (GEO) cohorts) were calculated by ssGSEA analysis. Subsequently, immune characteristic clustering was evaluated using R package and sparcl. All samples were clustered into two subgroups: Immunity-H group with an abundance of immune associated sets and Immunity-L group with an absence of immune associated sets. ESTIMATE algorithm was used to calculate the tumor purity and immune scores.

### TIMER2.0

TIMER2.0 (http://timer.cistrome.org/) (21) was utilized to investigate the correlations between VISTA expression and tumor purity, CD8 (+) T cell and M1 macrophage infiltration in basal-like BC.

### IHC and mIF

The clinical data and tumor pathological tissue samples of 16 patients with TNBC diagnosed in the First Affiliated Hospital of Xi’an Jiaotong University from October 2015 to October 2020 were retrospectively analyzed. According to standard IHC protocols, formalin-fixed paraffin-embedded (FFPE) tissue samples were immunohistochemically stained using VISTA antibody (D1L2G; dilution 1:200, Cell Signaling Technology, USA). The IHC scores of 0 to 4 were considered to be low expression of VISTA, while the scores of 5 to 12 were considered to be high expression of VISTA. Some markers were detected by mIF on PerkinElmer Mantra Quantitative Pathology Workstation/Quantitative Pathology Analysis Platform. The antibodies used included anti-panCK, anti-HLA-DR and anti-CD68. Previous studies used CD68 and HLA-DR for M1 identification (22–25). Hence, in this study, CD68 and HLA-DR double-positive cells were defined as M1 macrophages.

### Statistical Analysis

Survival analysis was performed using Kaplan–Meier method. Kruskal-Wallis and Wilcoxon tests were conducted to compare the expression difference of VISTA. Spearman’s or Pearson’s test was carried out for correlation analysis. All statistical analyses were completed by R (version 3.6.3) and R packages (https://www.r-project.org/). P < 0.05 was regarded to be statistically significant. All statistical tests were two-sided.

## Results

### Clinical significance of VISTA in TNBC

**Figure 1:**
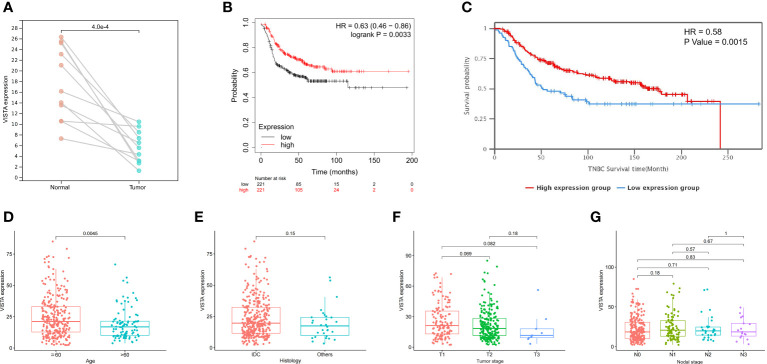
Correlations between VISTA expression and clinical parameters in TNBC patients. **(A)** Level of VISTA in paired tumor and normal tissues in TNBC based on TCGA database. **(B)** Survival curve using the Kaplan-Meier plotter is shown for RFS. **(C)** Kaplan-Meier survival curve of OS in the METABRIC database. Association of VISTA expression with clinicopathological characteristics, including age **(D)**, histology **(E)**, tumor stage **(F)** and nodal stage **(G)**. IDC, invasive ductal carcinoma.

To begin with, the data were downloaded from TCGA database to investigate the differences of the VISTA mRNA levels between paired tumor and normal tissues in TNBC. The results revealed that the VISTA levels in tumor tissues were significantly down-regulated (P = 0.0004; **Figure 1A**) compared with the adjacent normal tissues. Afterwards, the Kaplan-Meier Plotter database and METABRIC database were used to elucidate the relationship between VISTA expression and survival of TNBC patients. The results indicated that the high mRNA expression level of VISTA was significantly associated with better relapse-free survival (RFS) (hazard ratio (HR) = 0.63 (0.46-0.86), log-rank P = 0.0033 for 225373_at; **Figure 1B**) and OS (HR = 0.58, P = 0.0015; **Figure 1C**). Furthermore, the correlation between VISTA expression and the clinical parameters of TNBC was examined using FUSCC cohort. The results illustrated that younger patients (≦60 years) expressed remarkably higher VISTA level than patients over 60 years of age (P = 0.0045), while VISTA expression did not differ significantly according to histology, tumor stage and nodal stage (**Figures 1D-1G**). Collectively, the above results demonstrated that VISTA may serve as a potential biomarker for predicting survival in TNBC.

### Functional characteristics of VISTA in TNBC

**Figure 2:**
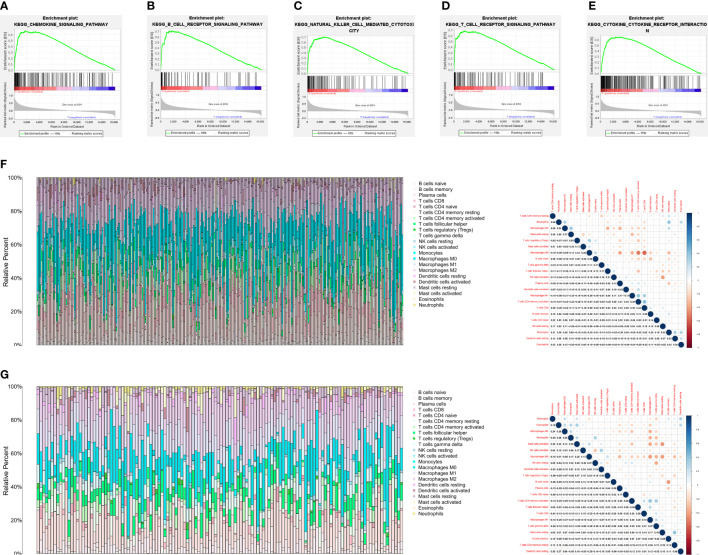
VISTA expression was markedly associated with tumor immunity. **(A-E)** The results of GSEA showed five VISTA-related signaling pathways. **(F)** The proportions of tumor immune infiltrating cell and the correlations between immune cells in FUSCC cohort. **(G)** The proportions of tumor immune infiltrating cell and the correlations between immune cells in GEO cohort. Each bar is an individual cancer sample.

After determining the prognostic value of VISTA in TNBC, GSEA was further performed using the TPM of RNA sequencing data from the FUSCC cohort to evaluate biological function and potential signaling pathway of VISTA. The results of Kyoto Encyclopedia of Genes and Genomes (KEGG) pathway enrichment analyses indicated that multiple immune-related pathways were mainly enriched in VISTA high expression phenotype of TNBC, including chemokine signaling pathway (normalized enrichment score (NES) =2.52, P < 0.001; **Figure 2A**), B cell receptor (BCR) signaling pathway (NES=2.47, P < 0.001; **Figure 2B**), natural killer (NK) cell-mediated cytotoxicity (NES=2.42, P < 0.001; **Figure 2C**), T cell receptor (TCR) signaling pathway (NES=2.41, P < 0.001; **Figure 2D**) and cytokine-cytokine receptor interaction (CCI) pathways (NES=2.39, P < 0.001; **Figure 2E**), etc. The 63 positively correlated KEGG pathways (false discovery rate (FDR) q-value < 0.05, NES > 1) were shown in **Supplementary Table 1**. Moreover, CancerSEA was used to assess the functional state of VISTA in BC. The results showed that VISTA expression exhibited a positive correlation with inflammation (Correlation = 0.69, P < 0.001), differentiation (Correlation = 0.60, P < 0.001), angiogenesis (Correlation = 0.43, P < 0.05), quiescence (Correlation = 0.38, P < 0.05) and metastasis (Correlation = 0.37, P < 0.05) in BC (**Supplementary Figure 1**). The CIBERSORT algorithm was further used to calculate the distribution of 22 immune cells in each TNBC sample. The proportions of tumor immune infiltrating cell (TIIC) and the correlations between immune cells in FUSCC and GEO cohorts were illustrated in **Figures 2F**, **G**, respectively.

### VISTA expression is correlated with immune infiltration in TNBC

**Figure 3:**
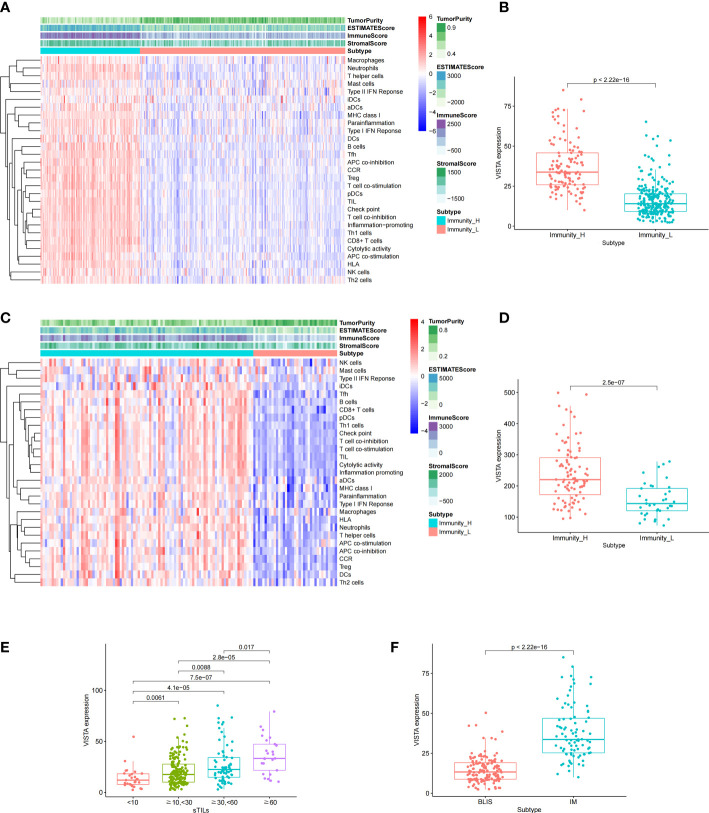
Based on the different gene expression of 29 pathways, TNBC was clustered into two main subtypes: Immunity_H and Immunity_L in FUSCC **(A)** and GEO **(C)** cohorts. Comparison of the expression level of VISTA between the two subtypes in FUSCC **(B)** and GEO **(D)** cohorts. Association of VISTA expression with clinicopathological characteristics, including stromal tumor infiltrating lymphocytes (sTILs) **(E)** and subtypes **(F)**.

Based on single sample GSEA algorithm, the enrichment scores of TNBC patients in FUSCC and GEO cohorts were calculated. The samples were classified into two subgroups with different immunity by unsupervised clustering. The Immunity_H group (FUSCC, 117 samples; GEO, 94 samples) was characterized with a high expression level of immune-related sets, and Immunity_L group (FUSCC, 242 samples; GEO, 37 samples) was performed with a low expression level of immune-related sets. At the same time, the immune scores (ESTIMATEScore, ImmuneScore, StromalScore) and tumor purity between two subgroups were assessed to confirm our group assignment with ESTIMATE algorithm. After comparison, it indicated that the Immunity_H group obtained a significantly higher immune scores and a remarkably lower tumor purity than Immunity_L group. Above all, the immunity in Immunity_H group was stronger than that in Immunity_L group (**Figures 3A, C**). In order to further explore the immunity of VISTA, the expression level of VISTA between Immunity_H and Immunity_L group was compared. The results showed that the expression level of VISTA in Immunity_H group was significantly higher than that in Immunity_L group (**Figures 3B, D**). Moreover, as the degree of sTILs infiltration increased, VISTA expression level gradually increased in TNBC (**Figure 3E**). Additionally, VISTA expression level was notably higher in the immunomodulatory (IM) subtype compared with the basal-like immune-suppressed (BLIS) subtype (**Figure 3F**).

**Figure 4:**
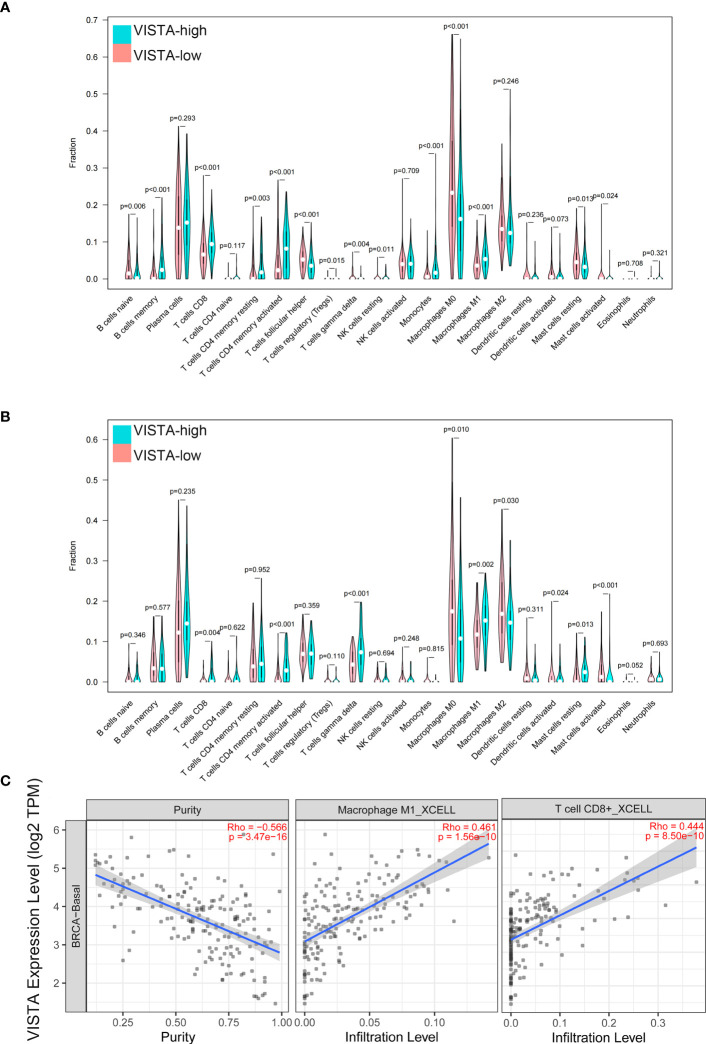
The immune cell infiltration difference between VISTA high and low groups in FUSCC **(A)** and GEO **(B)** cohorts. Correlation between VISTA expression and infiltration of M1 macrophage and CD8 (+) T cell **(C)** in basal-like BC.

In additional, the immune cell infiltration differences between VISTA high and low groups were analyzed. The results demonstrated that B cell memory (P < 0.001), T cell CD8+ (P < 0.001), T cell CD4 memory resting (P = 0.003), T cell CD4 memory activated (P < 0.001), T cell regulatory (Treg) (P = 0.015), monocytes (P < 0.001) and macrophage M1 (P < 0.001) exhibited a higher expression in the VISTA-high group, whereas B cells naive (P = 0.006), T cell follicular helper (P < 0.001), T cell gamma delta (P = 0.004), NK cells resting (P = 0.011), macrophage M0 (P < 0.001), mast cells resting (P = 0.013) and mast cells activated (P = 0.024) had a higher expression in the VISTA-low group in patients from the FUSCC cohort (**Figure 4A**). In order to ensure the accuracy of the results from the FUSCC cohort, the same analysis on the GEO cohort was performed. In agreement with the above results, T cell CD8+ (P = 0.004), T cell CD4 memory activated (P < 0.001) and macrophage M1 (P = 0.002) were upregulated, while macrophage M0 (P = 0.010) and mast cells activated (P < 0.001) were downregulated in the VISTA-high group (**Figure 4B**). What’s more, it was discovered VISTA expression exhibited a positive correlation with M1 macrophage and CD8 (+) T cell (**Figure 4C**) infiltration in basal-like BC using TIMER2.0.

### VISTA expression and the tumor immune microenvironment

**Figure 5:**
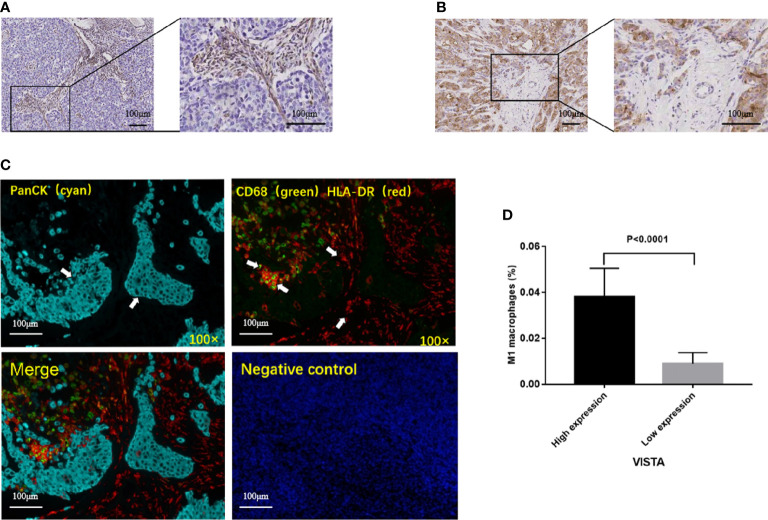
Representative immunohistochemical staining of VISTA, and correlation between VISTA expression and M1 macrophages in TNBC patients. **(A)** VISTA expression in stromal cells. **(B)** VISTA expression in tumor cells. **(C)** Multiplex immunofluorescence images from a TNBC patient. panCK (cyan); CD68 (green); HLA-DR (red); DAPI (blue). Cells were characterized as tumor cell (panCK (+)) and M1 macrophages (CD68 (+), HLA-DR (+)). **(D)** High protein level of VISTA in stromal cells was associated with higher infiltration of M1 macrophages.

The clinical samples in our hospital were collected. After obtaining institutional ethics committee approval and written informed consent, 16 TNBC patients were enrolled (**Supplementary Table 2**). The correlation between VISTA and immune cell infiltration was examined by IHC and mIF. The results reflected that the expression of VISTA in stromal cells (68.75%, 11/16) was higher than that in tumor cells (25%, 4/16). Only one case was positive for stromal cells and tumor cells, and one case was negative for both (**Figures 5A, B**). Most importantly, the high protein level of VISTA in stromal cells was associated with higher infiltration of M1 macrophages in TNBC (P < 0.0001) (**Figures 5C, D**).

## Discussion

TNBC is the most malignant subtype of BC, with limited treatment strategies, high incidence of tumor mutation burden (TMB) and tumor-infiltrating lymphocyte (TIL) rates. Phase III IMpassion130 study showed that atezolizumab combined with nab-paclitaxel improved the progression free survival (PFS) in patients with metastatic TNBC (3). Recent meta-analysis revealed that in metastatic BC, the pooled ORR of ICIs was 19% (95% CI = 12-27%), and TNBC had a relatively higher ORR (23%) than other BC subtypes (26). Although immunotherapy has achieved some success, only a few patients will benefit from it. Therefore, new immunotherapeutic targets are needed.

VISTA, a novel negative immune checkpoint, is a membrane protein with molecular weight of 55000 to 65000 daltons. It is highly conserved across different species, which suggested a conserved functional role (9). Previous study have found that TGF-β-induced Smad3 activation led to an increase in VISTA expression (27). In addition, hypoxia-inducible factor (HIF)-1α binding to a conserved hypoxia response element in the VISTA promoter could also upregulate VISTA on myeloid cells (28). The true functional binding partner(s) for VISTA is under investigation. At present, the potential ligands for VISTA include V-set and immunoglobulin domain 3 (VSIG-3), galectin-9 and so on (29). The engagement of VSIG-3 with VISTA can inhibit the proliferation of T cells and reduce their cytokine and chemokine release (30). Several studies have demonstrated that VISTA blockage has antitumour activity both *in vitro* and *in vivo*. VISTA inhibition was observed to decelerate tumour growth and increase survival rates in mouse models (13). At present, there are some clinical trials on VISTA. NCT02671955 and NCT02812875 are phase I clinical trials. India CTRI/2017/12/011026 is a phase II open label randomized trial, and its results show that the clinical benefit rate of CA-170 (inhibit PD-L1/PD-L2/VISTA) is 59.5% (31). The role of VISTA in controlling T cell activation is different from the PD-1/PD-L1 pathway (13). VISTA is increased after ipilimumab therapy in patients with prostate cancer (32) and anti-PD-1 therapy in patients with metastatic melanoma (33). This implied negative immune checkpoint regulation by VISTA is an important potential mechanism of acquired immunotherapy resistance. Moreover, Liju Zong et al. demonstrated a positive association between the levels of VISTA protein and PD-1/PD-L1 in breast cancer specimens (34). We found that the expression of VISTA was also positively correlated with the expression of PD-1 (**Supplementary Figure 2A**) and PD-L1 (**Supplementary Figure 2B**) in basal-like BC using TIMER2.0. The expression, prognostic significance and function of VISTA have been demonstrated in multiple tumor types, including melanoma, pleural mesothelioma, NSCLC, BC, pancreatic cancer, colorectal cancer, ovarian and endometrial cancer, and so on (31). However, the prognostic value and immunological role of VISTA in TNBC have not been extensively studied.

In this study, the expression of VISTA in paired TNBC tissues and matched normal tissues was analyzed from TCGA. The results indicated that VISTA was lowly expressed in TNBC tissues. Besides, it was found that VISTA was more highly expressed in younger patients with TNBC. Most importantly, survival curves revealed that VISTA was a good prognostic factor in TNBC, which was coincident with a previous report (35). A recent meta-analysis has also found that the high expression of VISTA was associated with significantly better OS (P < 0.001) (36). The above results suggested that VISTA could serve as a good prognostic factor for TNBC patients.

Subsequently, GSEA was performed using RNA sequencing data from the FUSCC cohort to evaluate the biological significance of VISTA in TNBC. The findings proved that among the top 10 statistically enriched KEGG pathways in TNBC patients with higher expression of VISTA, 5 pathways, including chemokine signaling pathway, BCR signaling pathway, NK cell-mediated cytotoxicity, TCR signaling pathway and CCI pathways, were related to immune function. In the relatively early stage of malignant tumor process, chemokines can induce lymphocyte infiltration, which may improve antitumor activity (37). For example, when CCR5 is expressed in T cells, it can enhance the antitumor response (38). BCR signaling plays a vital role at multiple checkpoints of B cell biology. BCR is necessary for B cells to correctly stimulate immune response (39). NK cells play a critical role in tumor immunosurveillance and tumor clearance. An 11-year follow-up survey conducted by Imai K illustrated that high NK cell cytotoxic activity was associated with reduced cancer risk (40). Tumor-infiltrating NK cells confer a positive prognostic value in colorectal carcinom, gastric carcinoma and squamous cell lung cancer (41). The recognition of cancer antigens by the TCR leads T cell activation, which inhibits tumor progression (42). CCI is closely related to immune reactions. Collectively, these results indicated that VISTA was closely related to immune infiltration in TNBC patients.

The Immunity_H group had a stronger immune signal. sTILs are the major immune defense against cancer cells, and associated with better prognosis in BC (43). A study conducted by Yi-Zhou Jiang classified TNBC into four transcriptome-based subtypes (1): luminal androgen receptor (2), IM, (3) BLIS, and (4) mesenchymal-like. Among them, IM subtype was characterized by high immune cell signaling. BLIS subtype was characterized by the down-regulation of immune response genes (18). In our study, it was revealed that the expression of VISTA was higher in the Immunity_H group and IM subtype. Moreover, higher expression of VISTA was remarkably associated with higher levels of sTILs. These findings suggested that VISTA expression was positively correlated with immunity in TNBC patients.

As we know, TIICs play a very vital role in tumor progression. CD8 (+) T cells have strong tumor killing ability. A study, involving 12,439 patients with BC, confirmed that CD8 (+) T cells were associated with good prognosis (44). Besides, several studies demonstrated that the expression of VISTA was positively correlated with CD8 (+) T cell infiltration. Xin-Lin He et al. retrospectively analyzed the medical records of 2440 patients. The results showed that the high expression of VISTA in solid tumours was strongly linked to better prognosis and high numbers of CD8 (+) TILs (P < 0.001) (36). Ming Zhang et al. also revealed there was a statistically significant positive association between VISTA and CD8+ mRNA expression in hepatocellular carcinoma, and VISTA+/CD8+ patients had a better OS (45). Similarly, our study found that CD8 (+) T cells exhibited a positive correlation with VISTA expression, which may partly explained why TNBC patients with high VISTA expression had a good prognosis.

Tumor associated macrophages (TAMs) are a major component of the TME. M0 macrophages are highly plastic. They can change their phenotype under the influence of environmental signals (46). Depending on the microenvironment, macrophages can mainly polarize into two functional phenotypes: classically activated macrophages (M1) and alternatively activated macrophages (M2) (47). M1 macrophages exert anti-tumor activity by releasing pro-inflammatory cytokines. The high density of M1 macrophages is associated with good prognosis in a variety of human malignancies, including NSCLC, HCC, ovarian and gastric cancers (48, 49). Besides, in the colorectal cancer, microsatellite instability-high tumors had higher densities of M1 macrophages in tumor stroma. M1 polarization of tumor stromal macrophages was related to lower cancer specific mortality (50). In our study, compared with VISTA-low group, VISTA-high group experienced significantly higher proportions of macrophage M1, as well as remarkable lower proportions of macrophage M0. The results suggested that VISTA-high group had a favorable immune microenvironment. IHC and mIF were further performed to validate the primary results that were revealed by bioinformatics analysis. The findings revealed VISTA level in stromal cells was positively correlated with M1 macrophages in TNBC, which was consistent with the above conclusion.

However, some potential limitations of our study should be noted. Our analysis showed that there was an association between the infiltration of CD8 (+) T cell and M1 macrophages and the expression of VISTA in TNBC, but the specific mechanism was unclear. Further studies are needed to verify the expression and function of VISTA *in vivo* and *in vitro*.

## Conclusion

In conclusion, our analysis suggested that VISTA was a good prognostic factor, and its expression was positively correlated with immunity in TNBC. Most importantly, the high level of VISTA was remarkably associated with high infiltration of CD8 (+) T cell and M1 macrophages. The results revealed that VISTA could be served as a potential biomarker for prognostic prediction and immune infiltration in TNBC.

## Data availability statement

The datasets presented in this study can be found in online repositories. The names of the repository/repositories or accession number(s) can be found below: https://portal.gdc.cancer.gov, The Cancer Genomic Atlas; https://www.ncbi.nlm.nih.gov/geo/, Gene Expression Omnibus, accession number GSE83937; http://kmplot.com/analysis, Kaplan-Meier plotter; http://www.omicsnet.org/bcancer/, Breast cancer integrative platform; http://biocc.hrbmu.edu.cn/CancerSEA/home.jsp, Cancer single-cell state atlas; http://timer.cistrome.org/, TIMER 2.0.

## Ethics statement

Written informed consent was obtained from the individual(s) for the publication of any potentially identifiable images or data included in this article.

## Author contributions

Research design: MZ, JZ and JY. Data collection and drafting: MZ, NL and BW. Statistical analysis: MZ, JZ and YZ. Manuscript polishing: MZ and JZ. Building figures: MZ and JZ. Manuscript editing: MZ and JY. Manuscript revision: MZ, JZ and JY. All authors have read and approved the manuscript. All authors contributed to the article and approved the submitted version.

## Acknowledgments

We would like to thank TCGA, GEO, CancerSEA, TIMER, METABRIC databases, and FUSCC for providing data.

## Conflict of interest

The authors declare that the research was conducted in the absence of any commercial or financial relationships that could be construed as a potential conflict of interest.

## Publisher’s note

All claims expressed in this article are solely those of the authors and do not necessarily represent those of their affiliated organizations, or those of the publisher, the editors and the reviewers. Any product that may be evaluated in this article, or claim that may be made by its manufacturer, is not guaranteed or endorsed by the publisher.

## Supplementary material

Supplementary Figure 1 Functional relevance of VISTA in BC. **(A)** Correlations between VISTA expression and 14 functional states in BC single-cell datasets. No.cells refers to the number of cells. **(B)** VISTA expression exhibited a positive correlation with inflammation, differentiation, angiogenesis, quiescence and metastasis in BC (***P < 0.001, **P < 0.01, *P < 0.05).

Supplementary Figure 2 Correlation between VISTA expression and PD-1 **(A)** and PD-L1 **(B)** expression in basal-like BC.
