# Supplementary material for: VISTA is associated with immune infiltration and predicts favorable prognosis in TNBC
Source: Front Oncol. 2022 Sep 8;12:961374. doi: 10.3389/fonc.2022.961374 (PMC9493462; doi:10.3389/fonc.2022.961374)
Supplement: Supplementary file 1 [file DataSheet_1.docx]

**Table S1** Results of GSEA.

| **Gene set name** | **NES** | **P-value** | **FDR q-value** |
| --- | --- | --- | --- |
| KEGG_CELL_ADHESION_MOLECULES_CAMS | 2.6746166 | < 0.001 | < 0.001 |
| KEGG_JAK_STAT_SIGNALING_PATHWAY | 2.5340047 | < 0.001 | < 0.001 |
| KEGG_CHEMOKINE_SIGNALING_PATHWAY | 2.5190268 | < 0.001 | < 0.001 |
| KEGG_B_CELL_RECEPTOR_SIGNALING_PATHWAY | 2.469621 | < 0.001 | < 0.001 |
| KEGG_NATURAL_KILLER_CELL_MEDIATED_CYTOTOXICITY | 2.4198248 | < 0.001 | < 0.001 |
| KEGG_T_CELL_RECEPTOR_SIGNALING_PATHWAY | 2.4169974 | < 0.001 | < 0.001 |
| KEGG_HEMATOPOIETIC_CELL_LINEAGE | 2.413927 | < 0.001 | < 0.001 |
| KEGG_CYTOKINE_CYTOKINE_RECEPTOR_INTERACTION | 2.3926022 | < 0.001 | < 0.001 |
| KEGG_INTESTINAL_IMMUNE_NETWORK_FOR_IGA_PRODUCTION | 2.392525 | < 0.001 | < 0.001 |
| KEGG_LEUKOCYTE_TRANSENDOTHELIAL_MIGRATION | 2.3529599 | < 0.001 | < 0.001 |
| KEGG_TOLL_LIKE_RECEPTOR_SIGNALING_PATHWAY | 2.3412712 | < 0.001 | < 0.001 |
| KEGG_LEISHMANIA_INFECTION | 2.3295681 | < 0.001 | < 0.001 |
| KEGG_SYSTEMIC_LUPUS_ERYTHEMATOSUS | 2.319279 | < 0.001 | < 0.001 |
| KEGG_VIRAL_MYOCARDITIS | 2.2940037 | < 0.001 | < 0.001 |
| KEGG_FC_GAMMA_R_MEDIATED_PHAGOCYTOSIS | 2.2438579 | < 0.001 | 5.25E-04 |
| KEGG_APOPTOSIS | 2.2232344 | < 0.001 | 4.93E-04 |
| KEGG_TYPE_I_DIABETES_MELLITUS | 2.2031546 | < 0.001 | 7.94E-04 |
| KEGG_RIG_I_LIKE_RECEPTOR_SIGNALING_PATHWAY | 2.2021673 | 0.002040816 | 7.50E-04 |
| KEGG_FC_EPSILON_RI_SIGNALING_PATHWAY | 2.1731117 | < 0.001 | 8.85E-04 |
| KEGG_GLYCOSPHINGOLIPID_BIOSYNTHESIS_GANGLIO_SERIES | 2.1586885 | < 0.001 | 9.57E-04 |
| KEGG_ANTIGEN_PROCESSING_AND_PRESENTATION | 2.1372676 | 0.002004008 | 0.001033266 |
| KEGG_AUTOIMMUNE_THYROID_DISEASE | 2.134146 | < 0.001 | 0.00113931 |
| KEGG_NOD_LIKE_RECEPTOR_SIGNALING_PATHWAY | 2.0780497 | < 0.001 | 0.001889977 |
| KEGG_PRIMARY_IMMUNODEFICIENCY | 2.0750797 | < 0.001 | 0.001856915 |
| KEGG_CYTOSOLIC_DNA_SENSING_PATHWAY | 2.0739253 | 0.002024292 | 0.00192343 |
| KEGG_ALLOGRAFT_REJECTION | 2.0523741 | 0.001992032 | 0.002807982 |
| KEGG_ACUTE_MYELOID_LEUKEMIA | 2.0469608 | < 0.001 | 0.003026755 |
| KEGG_MAPK_SIGNALING_PATHWAY | 2.0307534 | < 0.001 | 0.003466341 |
| KEGG_ASTHMA | 2.0239298 | 0.001964637 | 0.003643643 |
| KEGG_REGULATION_OF_ACTIN_CYTOSKELETON | 2.0193026 | < 0.001 | 0.00370654 |
| KEGG_TYPE_II_DIABETES_MELLITUS | 2.0090663 | < 0.001 | 0.003994412 |
| KEGG_ENDOCYTOSIS | 1.9965495 | < 0.001 | 0.004482822 |
| KEGG_COMPLEMENT_AND_COAGULATION_CASCADES | 1.9948103 | < 0.001 | 0.004448671 |
| KEGG_GRAFT_VERSUS_HOST_DISEASE | 1.984993 | 0.001949318 | 0.004937955 |
| KEGG_NEUROTROPHIN_SIGNALING_PATHWAY | 1.9569243 | < 0.001 | 0.006249042 |
| KEGG_NEUROACTIVE_LIGAND_RECEPTOR_INTERACTION | 1.9495802 | < 0.001 | 0.006376505 |
| KEGG_LYSOSOME | 1.9252666 | 0.010351967 | 0.007780833 |
| KEGG_ADIPOCYTOKINE_SIGNALING_PATHWAY | 1.9016514 | < 0.001 | 0.009663919 |
| KEGG_LONG_TERM_POTENTIATION | 1.898739 | < 0.001 | 0.009591733 |
| KEGG_PHOSPHATIDYLINOSITOL_SIGNALING_SYSTEM | 1.8931077 | 0.001984127 | 0.009810871 |
| KEGG_PATHWAYS_IN_CANCER | 1.8895462 | < 0.001 | 0.009809719 |
| KEGG_ALDOSTERONE_REGULATED_SODIUM_REABSORPTION | 1.8702794 | < 0.001 | 0.011417533 |
| KEGG_CALCIUM_SIGNALING_PATHWAY | 1.8405434 | < 0.001 | 0.014171287 |
| KEGG_MELANOMA | 1.8280768 | < 0.001 | 0.015886508 |
| KEGG_PANCREATIC_CANCER | 1.8149025 | 0.003898636 | 0.017160637 |
| KEGG_AMYOTROPHIC_LATERAL_SCLEROSIS_ALS | 1.8093331 | < 0.001 | 0.017611342 |
| KEGG_GLIOMA | 1.7842202 | 0.002040816 | 0.021513112 |
| KEGG_CHRONIC_MYELOID_LEUKEMIA | 1.7818626 | 0.002008032 | 0.021538977 |
| KEGG_SMALL_CELL_LUNG_CANCER | 1.7614595 | 0.004140787 | 0.02474694 |
| KEGG_MTOR_SIGNALING_PATHWAY | 1.7557285 | 0.004048583 | 0.025096018 |
| KEGG_NON_SMALL_CELL_LUNG_CANCER | 1.7488343 | < 0.001 | 0.02613101 |
| KEGG_FOCAL_ADHESION | 1.7429602 | 0.032786883 | 0.026984151 |
| KEGG_VEGF_SIGNALING_PATHWAY | 1.7311469 | 0.001976285 | 0.02858677 |
| KEGG_VASCULAR_SMOOTH_MUSCLE_CONTRACTION | 1.71363 | 0.01004016 | 0.03203562 |
| KEGG_PROSTATE_CANCER | 1.712213 | < 0.001 | 0.03169565 |
| KEGG_INSULIN_SIGNALING_PATHWAY | 1.6981905 | 0.01010101 | 0.03447013 |
| KEGG_NOTCH_SIGNALING_PATHWAY | 1.6935836 | 0.015936255 | 0.03529045 |
| KEGG_AXON_GUIDANCE | 1.6890657 | 0.002028398 | 0.035854623 |
| KEGG_SNARE_INTERACTIONS_IN_VESICULAR_TRANSPORT | 1.6869054 | 0.016194332 | 0.03597505 |
| KEGG_DORSO_VENTRAL_AXIS_FORMATION | 1.6624056 | 0.016161617 | 0.042254973 |
| KEGG_EPITHELIAL_CELL_SIGNALING_IN_HELICOBACTER_PYLORI_INFECTION | 1.6599509 | 0.01622718 | 0.042188264 |
| KEGG_ABC_TRANSPORTERS | 1.635592 | 0.026694044 | 0.047569774 |
| KEGG_COLORECTAL_CANCER | 1.6284332 | 0.011320755 | 0.048936162 |

NES, normalized enrichment score; FDR, false discovery rate.

**Table S2** Clinicopathological characteristics of 16 patients with TNBC collected from the First Affiliated Hospital of Xi'an Jiaotong University.

|  | **Age** | **Gender** | **VISTA** | **M1 macrophages (%)** |
| --- | --- | --- | --- | --- |
| Patient 1 | 29 | Female | High expression | 0.0538 |
| Patient 2 | 51 | Female | High expression | 0.0322 |
| Patient 3 | 59 | Female | High expression | 0.0258 |
| Patient 4 | 50 | Female | High expression | 0.0472 |
| Patient 5 | 35 | Female | High expression | 0.0455 |
| Patient 6 | 51 | Female | High expression | 0.0247 |
| Patient 7 | 60 | Female | Low expression | 0.0091 |
| Patient 8 | 42 | Female | Low expression | 0.0106 |
| Patient 9 | 41 | Female | Low expression | 0.0051 |
| Patient 10 | 53 | Female | Low expression | 0.0069 |
| Patient 11 | 36 | Female | Low expression | 0.0075 |
| Patient 12 | 43 | Female | Low expression | 0.0085 |
| Patient 13 | 63 | Female | Low expression | 0.0167 |
| Patient 14 | 63 | Female | Low expression | 0.0093 |
| Patient 15 | 66 | Female | Low expression | 0.0157 |
| Patient 16 | 48 | Female | Low expression | 0 |

VISTA, V-domain Ig-containing suppressor of T cell activation.
