# Supplementary figures and images for: VISTA is associated with immune infiltration and predicts favorable prognosis in TNBC
Source: Front Oncol. 2022 Sep 8;12:961374. doi: 10.3389/fonc.2022.961374 (PMC9493462; doi:10.3389/fonc.2022.961374)

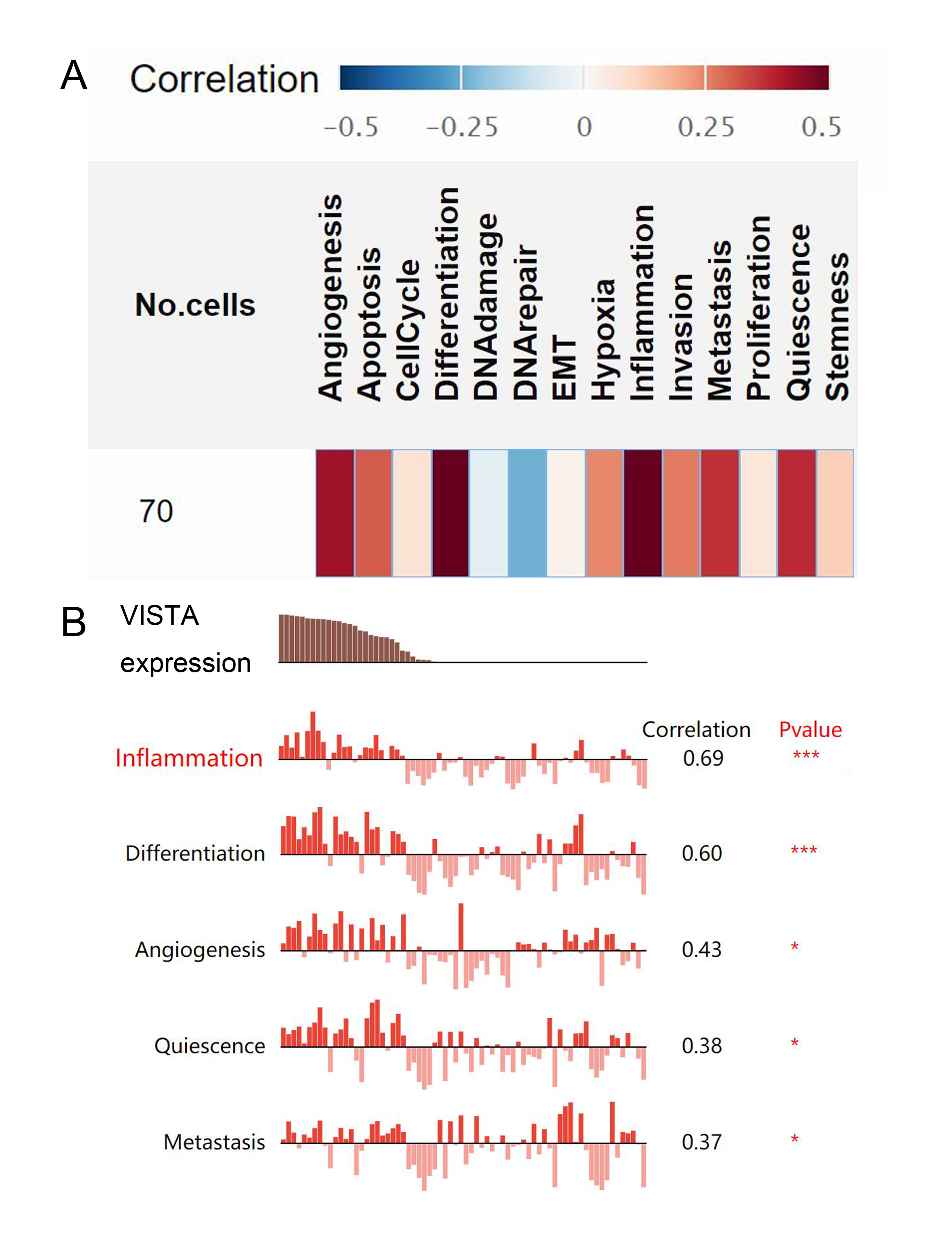

Supplement: Supplementary Figure 1 — Functional relevance of VISTA in BC. (A) Correlations between VISTA expression and 14 functional states in BC single-cell datasets. No.cells refers to the number of cells. (B) VISTA expression exhibited a positive correlation with inflammation, differentiation, angiogenesis, quiescence and metastasis in BC (***P < 0.001, **P < 0.01, *P < 0.05). [file Image_1.tif]

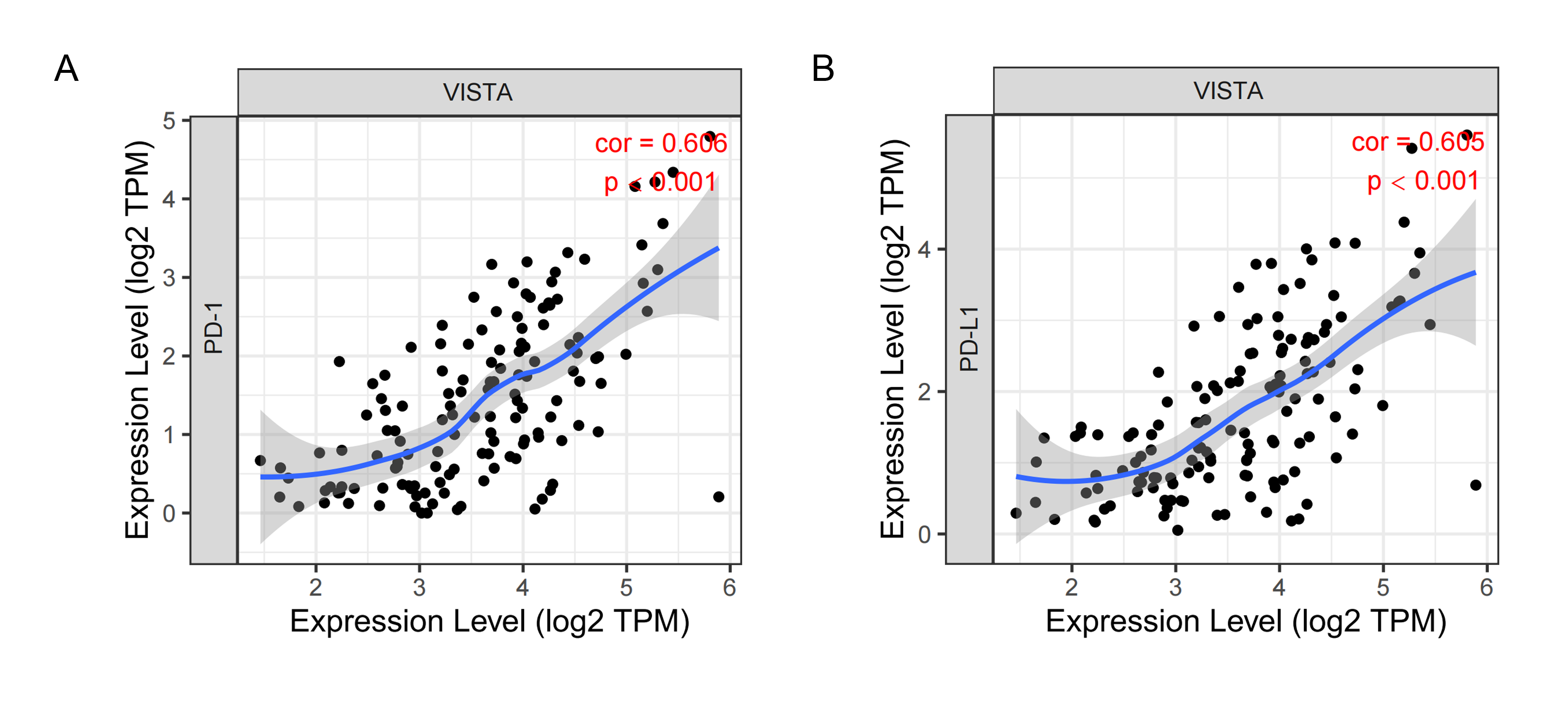

Supplement: Supplementary Figure 2 — Correlation between VISTA expression and PD-1 (A) and PD-L1 (B) expression in basal-like BC. [file Image_2.tif]
